# Trends in treatment for patients with depression in general practice in Norway, 2009–2015: nationwide registry-based cohort study (The Norwegian GP-DEP Study)

**DOI:** 10.1186/s12913-021-06712-w

**Published:** 2021-07-15

**Authors:** Sabine Ruths, Inger Haukenes, Øystein Hetlevik, Tone Smith-Sivertsen, Stefan Hjørleifsson, Anneli B Hansen, Sharline Riiser, Heidi Marie Meling, Valborg Baste

**Affiliations:** 1 Research Unit for General Practice, NORCE Norwegian Research Centre, Årstadveien 17, N-5009 Bergen, Norway; 2 Department of Global Public Health and Primary Care, University of Bergen, Bergen, Norway; 3 Division of Psychiatry, Haukeland University Hospital, Bergen, Norway

**Keywords:** Depression, General practice, Mental health, Drug therapy, Psychotherapy, Secondary care, Sick leave, Health services research, Large database research

## Abstract

**Background:**

Depression is highly prevalent, but knowledge is scarce as to whether increased public awareness and strengthened government focus on mental health have changed how general practitioners (GPs) help their depressed patients. This study aimed to examine national time trends in GP depression care and whether trends varied regarding patient gender, age, and comorbidity.

**Methods:**

Nationwide registry-based cohort study, Norway. The study population comprised all residents aged 20 years or older with new depression diagnoses recorded in general practice, 2009–2015. We linked reimbursement claims data from all consultations in general practice for depression with information on demographics and antidepressant medication. The outcome was type(s) of GP depression care during 12 months from the date of diagnosis: (long) consultation, talking therapy, antidepressant drug treatment, sickness absence certification, and referral to secondary mental health care. Covariates were patient gender, age, and comorbidity. The data are presented as frequencies and tested with generalized linear models.

**Results:**

We included 365,947 new depression diagnoses. Mean patient age was 44 years (SD = 16), 61.9 % were women, 41.2 % had comorbidity. From 2009 to 2015, proportions of patients receiving talking therapy (42.3–63.4 %), long consultations (56.4–71.8 %), and referral to secondary care (16.6–21.6 %) increased, while those receiving drug treatment (31.3–25.9 %) and sick-listing (58.1–50 %) decreased. The trends were different for gender (women had a greater increase in talking therapy and a smaller decrease in sick-listing, compared to men), age (working-aged patients had a smaller increase in talking therapy, a greater increase in long consultations, and a smaller decrease in antidepressant drug use, compared to older patients) and comorbidity (patients with mental comorbidity had a smaller increase in talking therapy and a greater increase in long consultations, compared to those with no comorbidity and somatic comorbidity).

**Conclusions:**

The observed time trends in GP depression care towards increased provision of psychological treatment and less drug treatment and sick-listing were in the desired direction according to Norwegian health care policy. However, the large and persistent differences in treatment rates between working-aged and older patients needs further investigation.

**Supplementary Information:**

The online version contains supplementary material available at 10.1186/s12913-021-06712-w.

## Background

Depression is among the three leading causes of years lived with disability (YLD) globally, at great personal and societal costs [1]. The ECNP/EBC Report estimated a one-year prevalence of major depression at 6.9 %, equivalent to 30.3 million people in Europe [2]. Women [3], older adults [4, 5], and people with multimorbidity [6, 7] are particularly at risk. A review of European community studies estimated that only 26 % of people with a mental disorder had sought health care, suggesting a considerable degree of unmet needs [8].

The World Health Organization (WHO) has recommended integrating mental health care into existing care settings to increase availability [9]. General practitioners (GPs) are usually the first encounter depressed people have with the health service, thus playing a crucial role in diagnosing and treating depression [10, 11]. In Norway, several government policies have been implemented that may change the provision of depression care in general practice. First, the National Program for Mental Health was implemented from 1999 to 2006 [12], mandating efforts to expand service capacity and quality in community-based and specialist mental health services for adults. The program resulted in a significant capacity shift from psychiatric beds to outpatient services [13]. With respect to primary care, low-threshold mental health services were established in the local communities, such as Healthy Life Centers, psychologists, outreach teams, and urgent mental health care. Second, a national guideline for diagnosing and treating adults with depression in primary and secondary care was published in 2009 [14]. While the use of antidepressant medication has increased dramatically during the 1990s [15, 16], the Norwegian and UK guidelines emphasize, e.g., the evidence base for drug treatment [14, 17]. Third, economic incentives are currently channeled into the field to stimulate GPs to give more priority to providing mental health care to their patients, e.g., talking therapy. Finally, policymakers have addressed the positive mental health effects of work participation, urging GPs to limit sick leave certification for patients with mild mental disorders [18]. Until now, however, little research has been conducted to ascertain whether and how GP depression care delivery has changed during the times of these policy changes.

The treatment of depression mainly consists of psychological and/or pharmacological interventions [19]. In moderate to severe cases, GPs can refer patients to secondary mental care provided by psychologists or psychiatrists. In some countries, including Norway, Sweden and the UK, GPs have an additional key role in certifying sick leave for patients with reduced work capacity [20]. Recent studies of GP depression care provided in the Netherlands and the UK indicate trends towards less prescription of antidepressant medication [21, 22]. However, time trends in GP depression care in Norway are poorly documented.

Based on information on all GP encounters and treatments stored in national databases, we examined the trends in treatment for depression in Norwegian general practice from 2009 to 2015. Further, we investigated whether trends varied across patient gender, age, and comorbidity.

## Methods

All residents in Norway have access to the public health services and prescription drugs (for example antidepressant drugs), covered by the National Insurance Scheme. A patient list-system was introduced in 2001, entitling all residents to have a regular GP [23]. The GPs provide comprehensive care for a broad range of health issues, and act as gatekeepers to secondary health care and social security benefits [24].

### Design

We conducted a nationwide registry-based cohort study comprising all individuals with one or more new depression diagnoses recorded in general practice during 2009–2015. We examined the provision of GP depression care for 12 months from the date of the depression diagnosis (index date).

### Data sources

Information drawn from national registries for the period 2008 through 2016 was linked at the individual patient level and GP level, using the (encrypted) unique personal identification number assigned to all residents of Norway. Data was stored and analyzed in a safe server at the University of Bergen.

The study population was drawn from the *Population Registry*. We obtained complete information regarding gender, year of birth, death, and emigration for all citizens born before 1989. The *Control and Reimbursement of Health Care Claims (KUHR) database* stores data on all fee-for-service claims from public primary care providers. For each encounter with a GP, we extracted information on date of contact, reimbursement code(s) for diagnostic and therapeutic measures, and one or more diagnoses according to the International Classification of Primary Care 2nd version (ICPC-2), as recorded by the GPs. *The Norwegian Prescription Database (NorPD)* contains information on all prescription drugs dispensed at pharmacies to individual patients treated in ambulatory care. For each prescription of a depression drug, NorPD provided information on date of dispensing, generic drug information (Anatomical Therapeutic Chemical (ATC) code), and any reimbursement code linked to specific diagnoses. NorPD lacks information at the individual level on medication dispensed to people staying in hospitals or nursing homes. The *Norwegian Patient Registry (NPR)* comprises information on all patient contacts with refundable secondary health care. We obtained information on date of depression contact with secondary care providers, with diagnoses according to the International Classification of Disease 10th revision (ICD-10). The data extracts provided by the national registries contained no missing information.

### Study population

The source population at risk comprised all residents of Norway born before 01.01.1996 and alive 01.01.2009 (4,017,989 individuals). First, we identified all individuals with one or more depression diagnoses recorded in ge-neral practice (GP-consultation with the ICPC-2 code P76 Depression in KUHR) during each year 2008–2015). Second, to establish a cohort of patients with *new* depression diagnoses, washout during 12 months *prior to index date* was performed for patients with a depression diagnosis in general practice (P76 in KUHR) or secon-dary health care (ICD-10 codes F32, F33, F34 or F41.2 in NPR), and/or dispensed antidepressant medication (NorPD). The 12-month contact-free interval was defined according to the algorithm published by Nielen and coworkers [25]. We thus identified 307,237 unique patients with new diagnoses of depression. Out of these, 51,753 incurred two or more depression episodes that were at least 12 months apart.

### Outcome

Information on GP depression care linked to ICPC-2 code P76 Depression, received during 12 months from the index date, was included from KUHR. We subsequently counted the number of GP consultations during one year from the index date. Types of depression care studied were consultations, including long consultations (i.e., > 20 min), talking therapy, referral to secondary mental health care, and certification of sickness absence. Throughout this paper, we use the term ‘talking therapy’ for GPs’ psychological treatment, including supportive talk, counseling, and structured psychotherapeutic methods such as cognitive-behavioral therapy [26–28]. Until July 2011, GPs could use the reimbursement code for talking therapy only if the patient had been referred to specialist mental health care, and until July 2014, this code could not be combined with the higher rewarding reimbursement code for long consultation. Considering that the latter change may have influenced how GPs use these reimbursement codes, we also provide combined figures. The variables were binary (yes, no). In addition, each variable had information on whether care started at the index consultation or if it started during follow-up. We investigated certification of sickness absence among patients between 20 and 66 years of age (at index date). From NorPD, we included all antidepressant drugs (ATC code N06A) reimbursed by the Norwegian State for the treatment of depression, dispensed during 12 months from the index date (yes, no).

### Covariates

Gender (women, men). We recoded patient age into two groups, 20–66 years (working-aged) and 67 + years. Pre-existing comorbidity was categorized as no pre-existing comorbidity, somatic only, mental only, or both somatic and mental health condition(s) [6].

### Statistical analysis

The annual incidence of patients recorded with a new depression diagnosis in general practice was calculated for gender and age groups, with the population aged 20 + as the denominator. Mean numbers of consultations with standard deviation (SD) were calculated for each year. In addition, consultation was divided into three groups (‘index date only’, ‘1–2’ or ‘3 or more’ follow-up consultations), and rates with a 95 % confidence interval (CI) were provided. Frequency of each type of GP depression care (talking therapy, long consultation, talking therapy and/or long consultation, referral to secondary care, sick leave certification among 20-66-year-olds) starting at index date, or after index date was calculated with a 95 % CI. Rate and 95 % CI of antidepressant drug treatment was established.

We defined a ‘GP-consultation-population’ comprising all GP consultations regardless of diagnosis, recorded in the KUHR database in the study period. We used this population to calculate the proportion of each type of treatment in consultations with a depression diagnosis and in consultations with other diagnoses, by year (panel a). For the study population, treatment rate and 95 % CI of each type of treatment, by year of depression diagnosis, were presented as graphs by gender (panel b), age group (panel c), and comorbidity (panel d). Antidepressant use in the general population [29] and in patients with depression was presented as graphs (panel a). Antidepressant use in the study population was also presented for each of the covariates (panel b-d).

For each treatment type, a generalized linear model (GLM) was used to test for linear time trends. Further, the time trend for each value of the covariate was tested, and in addition, the interaction between year and the covariate was tested. To evaluate the significance of the test results, a level of α = 0.001 was applied. We provide the results of all tests by *p*-values in Supplementary Table 1. The data were analyzed using STATA/SE version 16.1 (Stata Statistical Software).

## Results

**Fig. 1:**
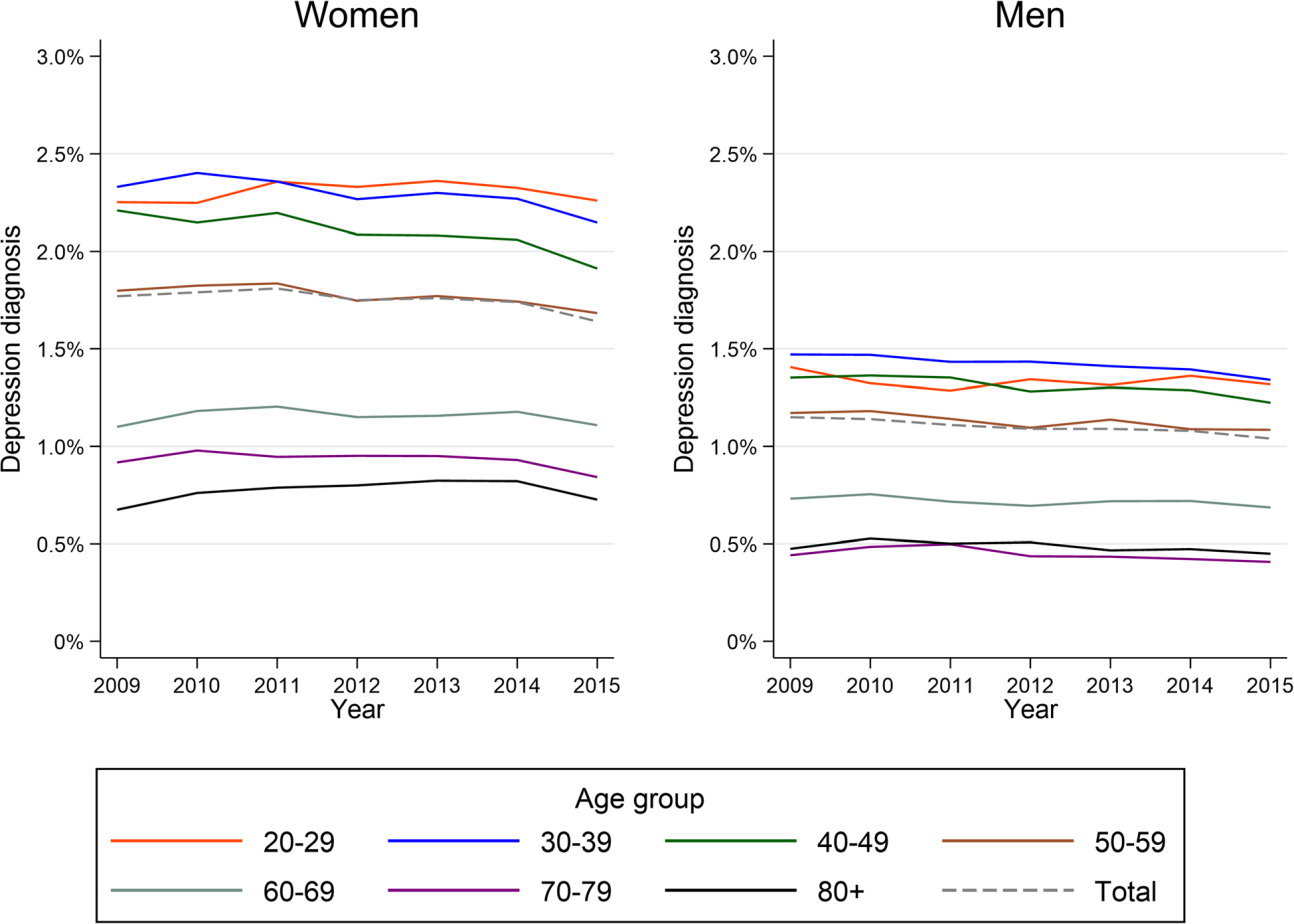
Annual incidence of patients recorded with a new depression diagnosis in general practice, by gender and age group

The study comprised 365,947 new depression diagnoses in 307,237 unique patients. Mean patient age at index date was 44.3 years (SD = 15.9; men: 43.7 (15.1); women: 44.6 (16.4)), and 61.9 % were women. Most patients were of working age (90.7 %) and 41.3 % had pre-existing comorbidity (27.0 % somatic, 9.3 % mental, 5.0 % both). The annual incidence of GP-recorded depression was 1.46 % in 2009 and decreased slightly to 1.34 % in 2015. For the whole period, the incidence was 1.8 % among women and 1.1 % among men. Figure 1 provides age- and year-specific depression incidence for genders separately.

The mean number of GP consultations for depression was stable throughout the study period; this also applied for having an index consultation only, 1–2 follow-up consultation(s), and 3 or more follow-up consultations, respectively (Table 1).

**Table 1:** Patients with a new depression diagnosis aged 20 years or older by year, and by GP depression care^a^ provided during 12-month follow-up

	Year of depression diagnosis													
	2009		2010		2011		2012		2013		2014		2015	
Number of patients with a new depression diagnosis	52,606		53,305		53,441		51,740		52,619		52,269		49,967	
	Mean	SD	Mean	SD	Mean	SD	Mean	SD	Mean	SD	Mean	SD	Mean	SD
Consultations per patient	3.41	3.21	3.41	3.25	3.38	3.22	3.40	3.25	3.41	3.27	3.43	3.27	3.46	3.30
Patients having:	%	95% CI	%	95% CI	%	95% CI	%	95% CI	%	95% CI	%	95% CI	%	95% CI
**Consultations**														
Index date	34.8	[34.4-35.2]	34.9	[34.5-35.3]	35.1	[34.7-35.5]	35.1	[34.7-35.5]	35.0	[34.7-35.4]	35.0	[34.5-35.4]	35.0	[34.5-35.4]
1-2 follow-up	32.2	[31.8-32.6]	32.5	[32.1-32.9]	32.8	[32.4-33.2]	32.5	[32.1-32-9]	32.8	[32.4-33.2]	32.4	[32.0-32.8]	32.0	[31.6-32.4]
3 or more follow-up	33.0	[32.6-33.4]	32.6	[32.2-33.0]	32.1	[31.7-32.5]	32.4	[32.0-32.8]	32.2	[31.8-32.6]	32.6	[32.2-33.0]	33.0	[32.6-33.4]
**Talking therapy**														
Index consultation^b^	27.0	[26.7-27.4]	31.2	[30.8-31.6]	37.6	[37.2-38.0]	38.0	[37.5-38.4]	39.7	[39.2-40.1]	43.5	[43.1-43.9]	51.1	[50.7-51.6]
Follow-up^c^	15.3	[15.0-15.6]	16.9	[16.5-17.2]	15.3	[15.0-15.6]	15.5	[15.2-15.8]	15.5	[15.2-15.8]	15.5	[15.2-15.8]	12.3	[12.0-12.6]
**Long consultation**^**d**^														
Index consultation ^b^	43.4	[43.0-43.8]	40.8	[40.4-41.2]	36.2	[35.7-36.6]	37.1	[36.7-37.5]	36.9	[36.5-37.3]	44.1	[43.7-44.5]	58.1	[57.7-58.5]
Follow-up^c^	13.0	[12.7-13.2]	13.0	[12.7-13.3]	13.9	[13.6-14.2]	13.9	[13.6-14.2]	15.5	[15.2-15.8]	16.7	[16.4-17.0]	13.7	[13.4-14.0]
**Talking therapy and/or long consultation**														
Index consultation ^b^	70.4	[70.1-70.8]	72.0	[71.6-72.4]	73.7	[73.4-74.1]	75.1	[74.7-75.4]	76.5	[76.2-76.9]	77.6	[77.3-78.0]	79.3	[78.9-79.6]
Follow-up^c^	10.7	[10.4-11.0]	10.3	[10.0-10.5]	9.3	[9.0-9.5]	9.1	[8.8-9.3]	8.9	[8.6-9.1]	8.5	[8.3-8.7]	7.9	[7.6-8.1]
**Referral to secondary care**														
Index consultation ^b^	8.1	[7.9-8.3]	8.8	[8.5-9.0]	8.3	[8.1-8.6]	9.5	[9.2-9.8]	10.1	[9.9-10.4]	10.9	[10.6-11.2]	11.6	[11.3-11.9]
Follow-up^c^	8.5	[8.3-8.8]	8.3	[8.0-8.5]	8.4	[8.1-8.6]	9.0	[8.7-9.2]	9.5	[9.3-9.8]	9.8	[9.6-10.1]	10.0	[9.8-10.3]
**Antidepressant drugs**	31.3	[30.9-31.7]	30.4	[30.0-30.8]	29.7	[29.3-30.1]	28.2	[27.8-28.6]	26.3	[25.9-26.7]	26.9	[26.5-27.2]	25.9	[25.5-26.2]
**Sick leave certification**^**e**^														
Index consultation ^b^	47.5	[47.1-48.0]	42.9	[42.4-43.3]	42.1	[41.7-42.6]	40.9	[40.5-41.3]	39.8	[39.4-40.3]	38.8	[38.4-39.3]	39.2	[38.7-39.6]
Follow-up^c^	10.6	[10.4-10.9]	10.9	[10.7-11.2]	10.5	[10.3-10.8]	10.7	[10.4-10.9]	10.8	[10.5-11.0]	10.9	[10.6-11.2]	10.8	[10.5-11.1]

^a^ GP depression care: consultations and initiatives related to P76 Depression. Patients may have received more than one treatment option

^b^ Treatment started at the index consultation and may have continued during follow-up consultation(s)

^c^ Treatment started after the index consultation

^d^ Long consultation = lasting > 20 min

^e^ Patients aged 20–66 years

**Fig. 2:**
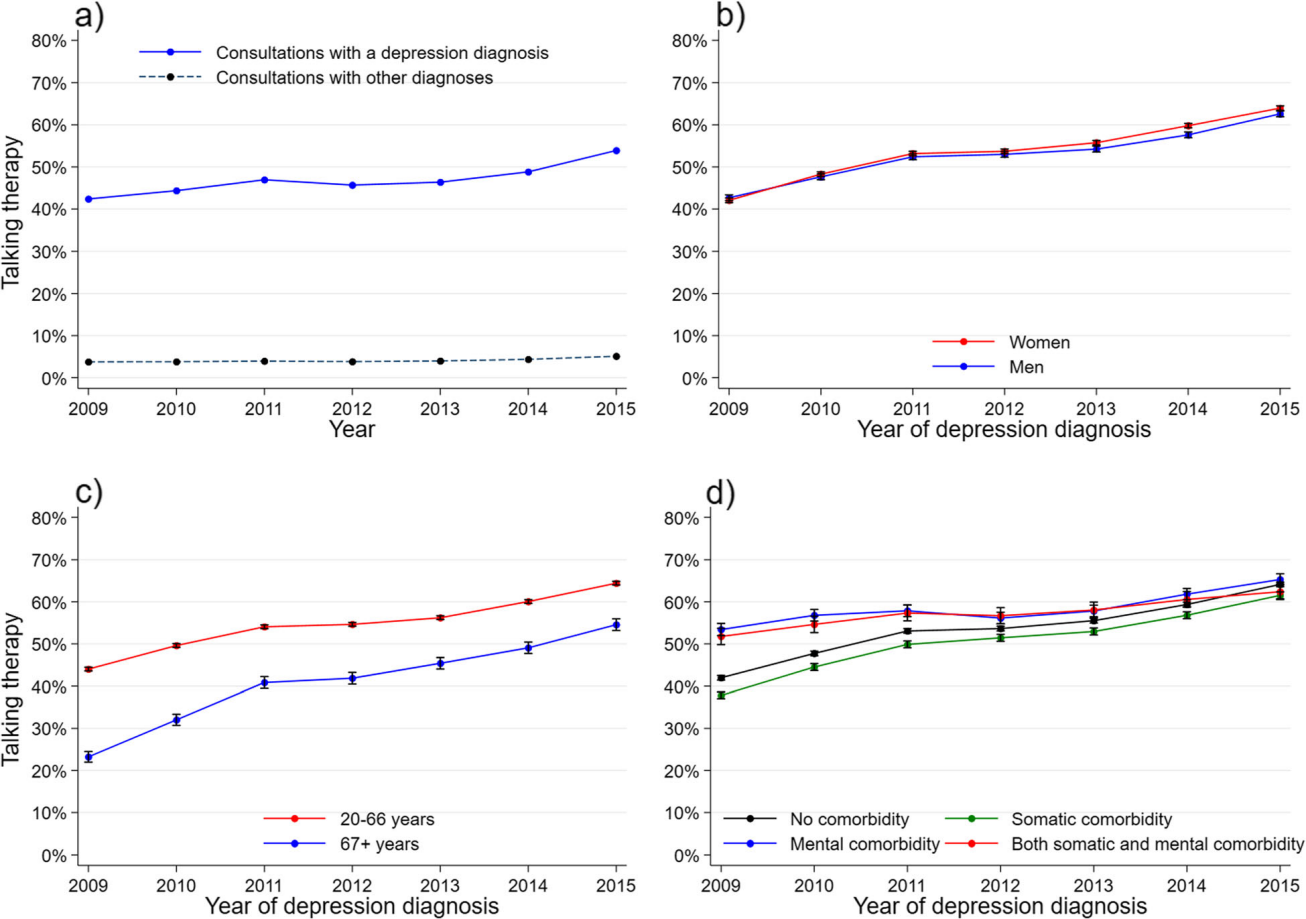
Talking therapy by year. **a** GP population: proportion of talking therapy in consultations with a depression diagnosis, and consultations with other diagnoses. **b-d** Study population: treatment rate and 95% CI during one-year follow up by **b** gender, **c** age group and **d** comorbidity

The provision of talking therapy in a consultation increased during the study period (Fig. 2a). In terms of treatment rates for depression, talking therapy starting at index consultation increased from 27.0 % to 2009 to 51.1 % in 2015, while talking therapy starting during follow-up was stable over the years (Table 1). Women had a larger increase in talking therapy provision compared to men (Fig. 2b). A larger proportion of working-aged patients had talking therapy with the GP compared to patients aged 67+. However, the latter group had a greater increase in talking therapy during the first few years than the younger patients (Fig. 2c). Patients without comorbidity or with somatic comorbidity received less talking therapy early in the study period and gradually more in later years, reaching a similar level as those with comorbid mental conditions (Fig. 2d).

**Fig. 3:**
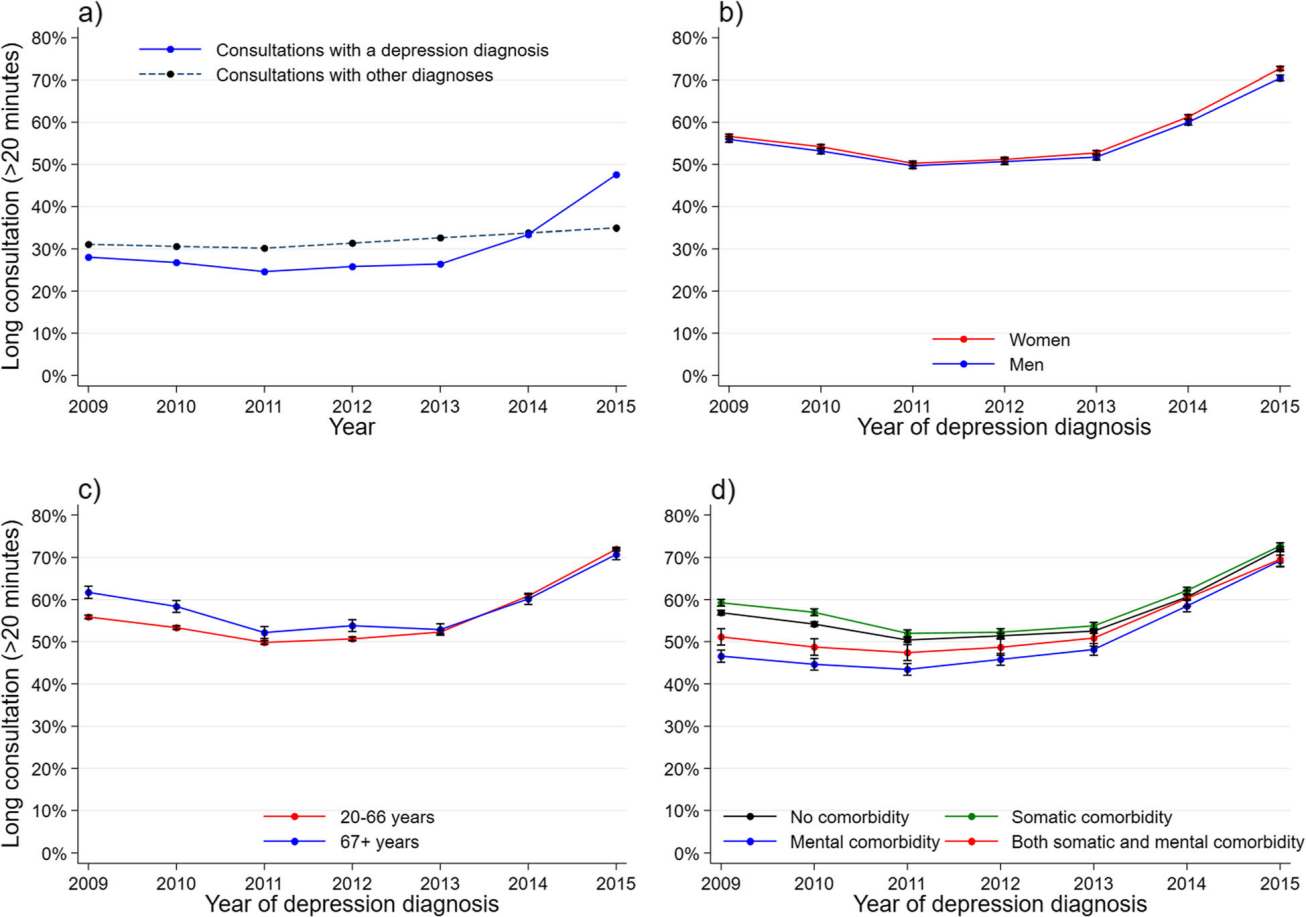
Long consultation (> 20 minutes) by year. **a** GP population: proportion of long consultations (> 20 minutes) in consultations with a depression diagnosis, and consultations with other diagnoses. **b-d** Study population: treatment rate and 95% CI during one-year follow up by **b** gender, **c** age group and **d** comorbidity

Long consultations (i.e., > 20 min) increased during the study period, and the increase was pronounced from 2013 to 2015 for consultations with a depressed patient (Fig. 3a). For patients with a new depression diagnosis, the rates of long consultations starting at index consul-tation increased mainly in the last two years of the period, while long consultations starting during follow-up were stable (Table 1). The time trends for long consultations were similar for genders (Fig. 3b). Patients aged 67 + had higher rates of long consultations from 2009 to 2012, and from 2013, both age groups had increasing rates (Fig. 3c). Those with somatic comorbidity or without comorbidity had higher rates of long consultations compared to those with mental comorbidity, but differences became less pronounced towards the end of the study period (Fig. 3d).

The combination of talking therapy and/or long consultation starting at index consultation increased gradually over the years (Table 1).

**Fig. 4:**
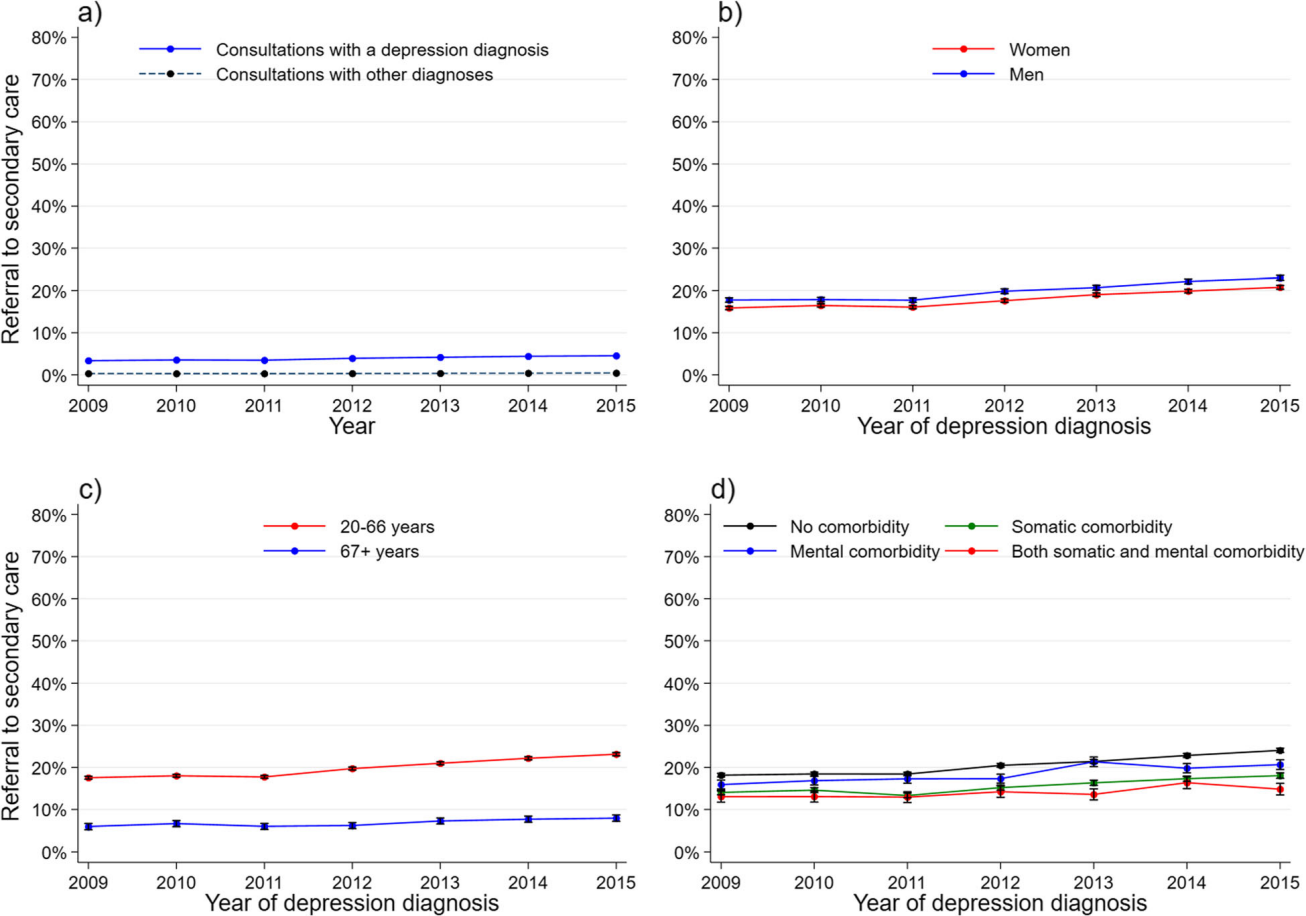
Referral to secondary care by year. GP population: proportion of referral to secondary care in consultations with a depression diagnosis, and consultations with other diagnoses. **b-d** Study population: treatment rate and 95% CI during one-year follow up by **b** gender, **c** age group and **d** comorbidity

There was a weak increase over the years to refer a patient to specialist mental health care in a consultation, both for depressed patients and patients with other diagnoses (Fig. 4a). For patients with depression, the referral rates at index date and during follow-up increased gradually (Table 1). Men, working-aged patients, and those without comorbidity were more commonly referred compared to women, those aged 67+, and patients with comorbidity, respectively (Fig. 4b and d). However, there were no interactions between year and any of the covariates.

**Fig. 5:**
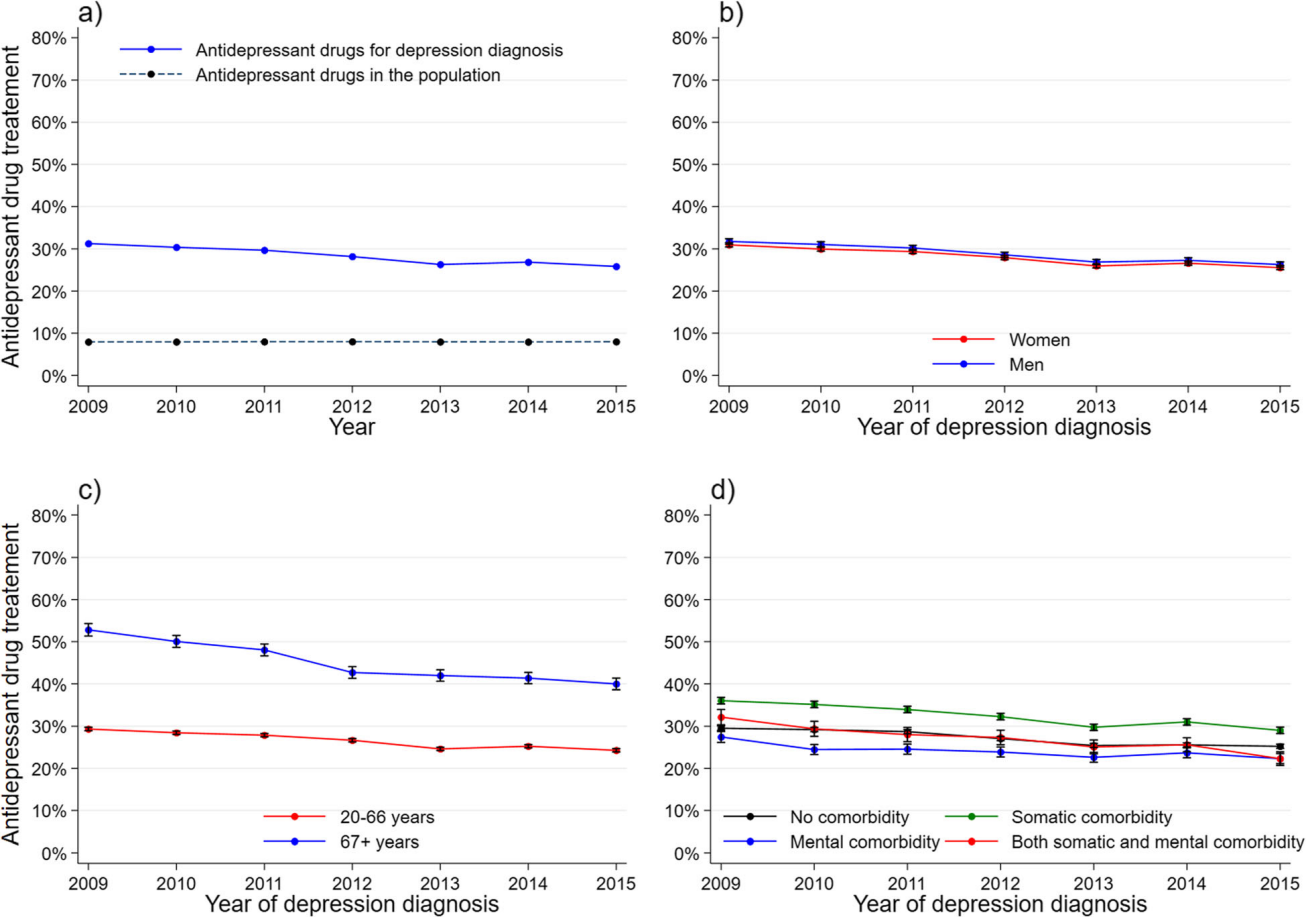
Antidepressant drug treatment by year. **a** Proportion of antidepressant drug use among patients with a depression diagnosis, and of the general population aged 20+. **b-d** Study population: treatment rate and 95% CI during one-year follow up by **b** gender, **c** age group and **d** comorbidity

The proportion of depressed patients who were dispensed antidepressant drugs decreased from 31.3 % to 2009 to 25.9 % in 2015 (Table 1; Fig. 5a). Men had slightly higher treatment rates compared to women, but the time trends were similar for genders (Fig. 5b). For patients aged 67+, the decrease in antidepressant use was more pronounced (from 52 % to 2009 to 40 % in 2015) than for working-aged patients (Fig. 5c). Drug treatment decreased among all categories of comorbidity, and the trends were similar (Fig. 5d).

**Fig. 6:**
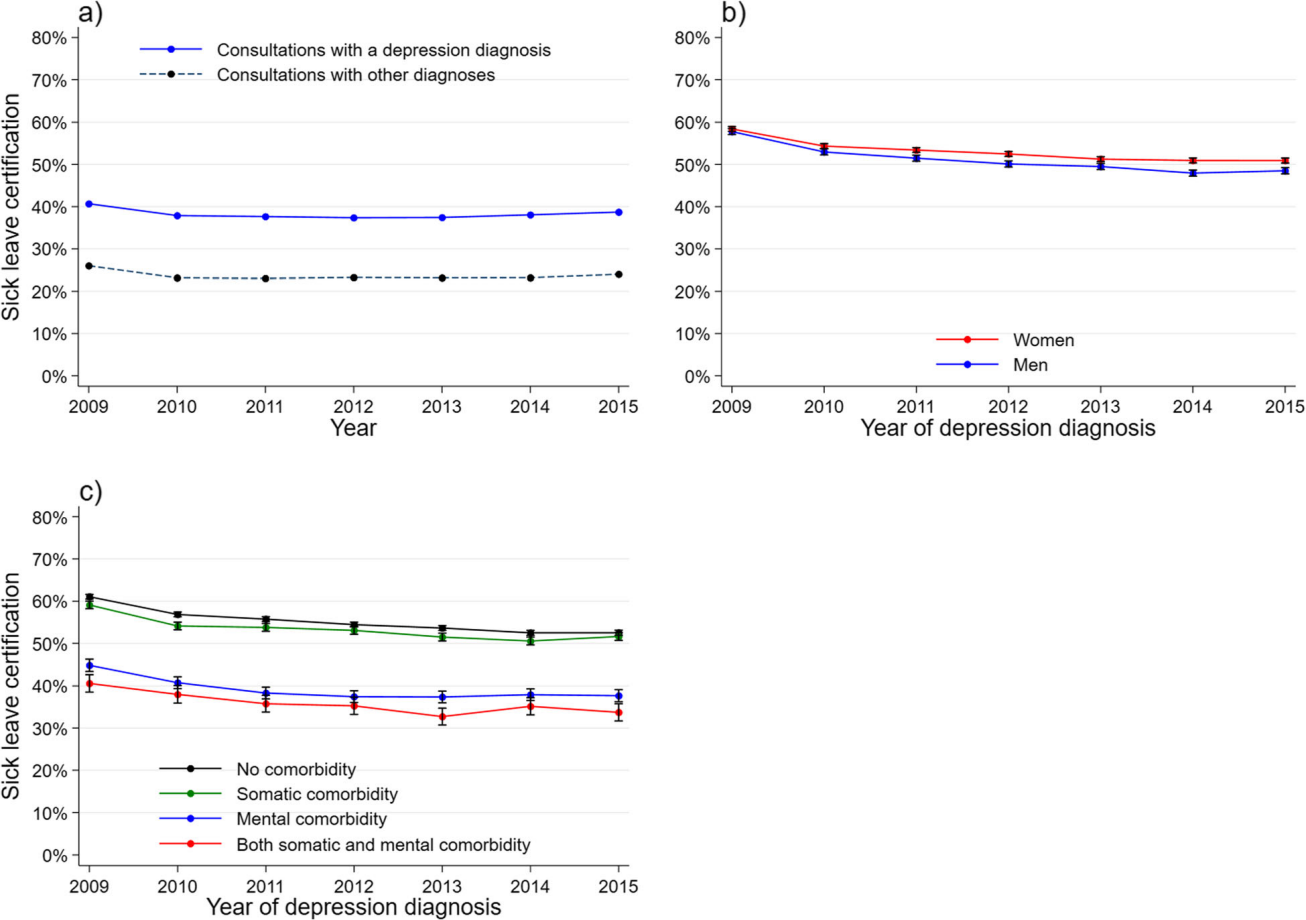
Sick leave certification among patients 20-66 years old, by year. **a** GP population: proportion of sick leave certification in consultations with a depression diagnosis, and consultations with other diagnoses. **b-c** Study population: treatment rate and 95% CI during one-year follow up by **b** gender and **c** comorbidity

Sick-listing by GP, regardless of diagnosis, decreased from 2009 to 2010 (Fig. 6a). For depressed patients, sick leave certified at index consultation decreased from 47.5 % to 2009 to 39.2 % in 2015. About 10 % of the patients were sick-listed during follow-up, and this percentage did not change throughout the period (Table 1). A greater share of women than men was sick-listed, and the decrease was more pronounced in men over the years (Fig. 6b). There was a reduction over time in sick leave certification for patients without comorbidity or with somatic comorbidity only. For those with mental comorbidity, sickness absence certification was lower than for other groups but did not change over time, and there was no interaction between year and comorbidity (Fig. 6c).

## Discussion

### Summary

In a nationwide cohort of patients with one or more new depression diagnoses, we examined time trends in depression care provided by the GP from 2009 to 2015. The one-year incidence rates of depression recorded in general practice decreased slightly during the study period. With respect to type of GP care, we found trends towards more provision of talking therapy, more long consultations, and more referral to secondary care, whereas drug treatment and sick-listing decreased over time. The time trends were different for gender (women had a greater increase in talking therapy and a smaller decrease in sick-listing, compared to men), age (working-aged patients had a smaller increase in talking therapy, a greater increase in long consultations, and a smaller decrease in antidepressant drug use, compared to older patients) and comorbidity (patients with mental comorbidity had a smaller increase in talking therapy and a greater increase in long consultations, compared to no comorbidity and somatic comorbidity).

### Strengths and limitations

The main strength of this study is the use of complete registry data from the publicly subsidized primary care services in Norway. Linked data from five national health- and population registries at the individual patient level provides a unique source of information, elimina-ting recall bias.

We defined a new depression diagnosis as a GP-consultation with the ICPC-2 code P76, after a one-year wash-out. However, our population was restricted to patients whose depression was identified by the GP and recorded as such. Patients with depression who did not seek help or were not diagnosed with depression by the GP remained beyond our scope. Further, information on the severity of depression was lacking, as ICPC-2 does not allow for such grading.

A limitation of using the reimbursement code for talking therapy was that the prerequisites were changed during the study period. The recorded reimbursement codes probably did not catch all performed GP talking therapy sessions, because referral to secondary mental care was a requirement until July 2011, and until July 2014, this code could not be combined with the higher rewarding reimbursement code for a long consultation (> 20 min).

The NorPD contains complete data on all prescription drugs *dispensed*, thus we may have slightly underestimated the prevalence of *prescribed* antidepressants. To strengthen the internal validity, we have considered antidepressants reimbursed for the treatment of depression only. No information was available on the appropriateness of the different types of treatment provided.

The results of this study could be transferable to countries where GPs fulfill comparable roles to the Norwegian GPs (having fixed, personalized patient lists, and acting as gatekeepers), such as Sweden, the UK, and the Netherlands. They are less comparable with countries where GPs have different task assignments.

### Interpretation of findings and comparison with existing literature

#### Patient population recorded with depression in general practice

The gender distribution in our study population is consistent with previous research [30], reflecting a higher prevalence of depression [3] and more doctor-seeking [31] among women compared to men. Although older people are at greater risk of depression [4, 5], we found that the annual incidence of GP-recorded depression decreased with increasing age (Fig. 1). This may reflect that the course of depression becomes more prolonged / chronic with increasing age. Also, older people are less inclined to seek help for their psychological problems [32].

The decrease in annual incidence rate of GP-recorded depression is consistent with trends reported in the UK [33]. This change occurred in the context of increasing availability of low-threshold mental health services in the local communities and self-guided internet-based interventions. This raises the question whether people have changed their health care-seeking behavior, i.e., those with more severe depression seek their GP while those with less severe depression use the newly established low-threshold services instead. However, the Norwegian health registries do not include information at the individual level about the provision of low-threshold services for depressed people. Further, the observed time trend to refer more patients, coinciding with increasing capacity in outpatient secondary mental care [13], was likely attributable to a selection of patients with more severe depression (in need of referral) and/or GPs’ altered referral behavior.

#### Time trends in GP-depression care

Although there was a substantial increase in long consultations, the mean number of GP follow-up consultations did not change over time. In comparison, numbers of GP contacts for depression in the Netherlands increased after implementing a stepped collaborative depression programme [34] and integra-ting mental health nurses in primary care teams [35]. So far, however, such targeted initiatives have not been implemented in Norway.

Our findings indicate that provision of talking therapy increased, while antidepressant drug treatment decreased over time. These changes occurred following the publication of national guidelines that encourage improved targeting of drug treatment for depression and emphasize psychological treatment as GPs’ primary tool in depression care [14]. Further, GPs’ increasing provision of talking therapy and long consultations during the last years of the study was likely attributable (in part) to a change in the terms in mid-2014, i.e., both tariffs could be used together. Finally, patient preferences may be drivers of change as patients increasingly participate in the decision-making about their health care [36]. Notably, a meta-analytic review across different settings yielded a 70 % greater patient preference for psychological treatment than for pharmacological treatment for depression [37].

From an international perspective, GPs’ antidepressant prescribing rates in the Netherlands (2011–2015) decreased more than in Norway [21], while prescribing rates in the UK (2003–2013) declined only for first-ever depression episodes [22]. In US outpatient treatment of depression (2007–2015), the use of psychotherapy increased, while drug treatment remained stable [38]. Generally, the 26-31 % antidepressant prescribing rates found in this study are considerably lower than the 45-75 % rates reported in European countries with comparable primary care systems like the UK, the Netherlands, and Sweden [21, 22, 30]. This discrepancy may be due to the strict definitions of antidepressant drugs (excluding prescriptions issued for other diagnoses than depression) and drug dispensing data in this study.

The observed decrease over time in GPs’ sick leave certification was attributable to less sick-listing starting at index date. This trend is in accordance with Norwegian government policy [18] and European policy [39], urging GPs to limit certification of sick leave in patients with mild mental disorders. Studies indicate that mental disorders have gradually replaced musculoskeletal problems as the main cause of sickness certification [40], with depression as the leading cause of GPs’ sickness certification related to common mental disorders [41]. Possibly, intensified GP depression care in terms of more talking therapy and long consultation have contributed to reducing sickness absence [42].

#### Time trends related to patient gender, age, and comorbidity

Men were slightly more likely to receive drug treatment and slightly less likely to be sick-listed, compared to women, consistent with previous research [29, 41, 43]. The greater proportion of women than men receiving talking therapy or long consultations with the GP, and the greater increase in talking therapy among women may be related to gendered patient preferences, i.e., women express and talk about their feelings more easily [44]. Greater referral rates and antidepressant prescription rates among men compared to women, may relate to less doctor-seeking [31], and more severe depression at diagnosis date.

The greater increase among older patients compared to working-aged patients has reduced but not eliminated the age gap regarding the provision of talking therapy. Also, referral rates for depressed patients aged 67 + were persistently lower than for working-aged patients, in line with findings in a comparable study in the UK [45]. An explanation of the observed age gap in psychological treatment in general practice and referral to secondary care may be that older people wish to solve their emotional problems autonomously and fear of stigma [46]. However, a systematic review of qualitative studies indicates that older people express preferences for talking therapies [47]. Further, the considerably higher antidepressant prescription rates for older patients than working-aged patients observed in this study are consistent with the beforementioned study in the UK [45]. Although the drug treatment rate for older patients decreased over time, it remained significantly higher than for patients of working age. This is of concern because antidepressants are associated with adverse events, such as falls and stroke, in older people [48].

The higher antidepressant treatment rate in patients with somatic comorbidity compared to those with mental comorbidity or without comorbidity was a surprising finding. Comparable studies of patients newly diagnosed with depression in general practice revealed higher prescription rates for Dutch patients with chronic somatic or psychiatric comorbidity [49], while prescription rates in the UK were lower for patients with comorbid so-matic illness [50], compared to those without comorbidity. Smolders et al. point out that GPs have higher contact rates with patients who suffer from multimorbidity and thus have more opportunities to treat their depression [49]. Further, our findings indicate that patients without comorbidity or with somatic comorbidity were more easily sick-listed than those with mental comorbidity. This difference may be attributable to patients with several mental conditions having more long-term periods of sick-leave, i.e., being not at risk of being sick-listed when a new depression episode starts. Also, GPs may have limited sick-listing of patients with mild mental disorders, in line with Norwegian [18] and European po-licy [39].

### Implications for research and/or practice

The European Mental Health Action Plan 2013–2020 emphasized the importance of integrating mental health care into the general health care setting [9]. Worldwide, many initiatives have been undertaken to promote the substitution of mental health care from specialized care, e.g., provision of psychological treatment by the GP [27, 28]. Our findings support that most patients diag-nosed with depression by the GP are treated within general practice, in line with previous research [11]. The provision of talking therapy and long consultations increased substantially, in accordance with government policy in Norway. However, the average number of GP consultations for depression was unchanged. This may point at sustainability is being challenged because general practice as a service level has not been strengthened through the National Program for Mental Health [12] or other recent reforms.

## Conclusions

The observed time trends in GP depression care towards more provision of psychological treatment, and less provision of pharmacological treatment and sick-leave certification were in the desired direction according to Norwegian health care policy. However, the large and persistent differences in treatment rates between working-aged and older patients is of concern. We have planned follow-up studies to examine the reasons for these differences. Further, clinical studies are needed to explore the implications of the observed changes for patient outcomes.

## Supplementary Information

**Additional file 1.**

## Data Availability

The data underlying this article were provided by the Norwegian Directorate of Health, the Norwegian Institute of Public Health, and Statistics Norway by permission. The data cannot be shared publicly due to restrictions by the Norwegian Data Protection Authority.

## Abbreviations

ATC: Anatomical Therapeutic Chemical
CI: Confidence interval
GLM: Generalized Linear Model
GP: General practitioner
ICD: International Classification of Disease
ICPC: International Classification of Primary Care
KUHR: Control and Reimbursement of Primary Health Care Claims Database
NorPD: Norwegian Prescription Database
NPR: Norwegian Patient Registry
WHO: World Health Organisation
YLD: Years lived with disability
