# Supplementary material for: Trends in treatment for patients with depression in general practice in Norway, 2009–2015: nationwide registry-based cohort study (The Norwegian GP-DEP Study)
Source: BMC Health Serv Res. 2021 Jul 15;21:697. doi: 10.1186/s12913-021-06712-w (PMC8283991; doi:10.1186/s12913-021-06712-w)
Supplement: Supplementary file 1 — Additional file 1. [file 12913_2021_6712_MOESM1_ESM.docx]

**Supplementary Table. Provision of GP depression care in Norway (2009-2015). Test for linear time trend and interaction between year and the covariates, provided as p-value (generalized linear model, GLM).**

|  |  |  | | |  |  | | |  |  | | |  |  | | |  |  | | | |  |  | | |  |
| --- | --- | --- | --- | --- | --- | --- | --- | --- | --- | --- | --- | --- | --- | --- | --- | --- | --- | --- | --- | --- | --- | --- | --- | --- | --- | --- |
|  |  | **Type of GP depression care** | | | | | | | | | | | | | | | | | | | | | | | | |
| **Covariates** |  | **Consultation** | | |  | **Talking therapy** | | |  | **Long consultation** | | |  | **Referral to secondary care** | | |  | **Antidepressant drugs** | | | |  | **Sick leave certification** | |  |  |
|  |  |  | | |  |  | | |  |  | | |  |  | | |  |  | | | |  | |  |  |  |
| **Year** |  | .62 | | |  | <.0001 | | |  | <.0001 | | |  | <.0001 | | |  | <.0001 | | | |  | | <.0001 |  |  |
|  |  |  | | |  |  | | |  |  | | |  |  | | |  |  | | | |  | |  |  |  |
| **Gender** |  |  | | |  |  | | |  |  | | |  |  | | |  |  | | | |  | |  |  |  |
| Male |  | .06 | | |  | <.0001 | | |  | <.0001 | | |  | <.0001 | | |  | <.0001 | | | |  | | <.0001 |  |  |
| Female |  | .39 | | |  | <.0001 | | |  | <.0001 | | |  | <.0001 | | |  | <.0001 | | | |  | | <.0001 |  |  |
| Interaction |  | .044 | | |  | <.0001 | | |  | .0051 | | |  | .98 | | |  | .94 | | | |  | | .0001 |  |  |
|  |  |  | | |  |  | | |  |  | | |  |  | | |  |  | | | |  | |  |  |  |
| **Age** |  |  | | |  |  | | |  |  | | |  |  | | |  |  | | | |  | |  |  |  |
| 20-66 |  | .79 | | |  | <.0001 | | |  | <.0001 | | |  | <.0001 | | |  | <.0001 | | | |  | |  |  |  |
| 67+ |  | .34 | | |  | <.0001 | | |  | <.0001 | | |  | <.0001 | | |  | <.0001 | | | |  | |  |  |  |
| Interaction |  | .32 | | |  | <.0001 | | |  | <.0001 | | |  | .85 | | |  | <.0001 | | | |  | |  |  |  |
|  |  |  | | |  |  | | |  |  | | |  |  | | |  |  | | | |  | |  |  |  |
| **Comorbidity** |  | |  |  | | |  |  | | |  |  | | |  |  | | |  |  |  |  |  |  |  |  |
| None |  | .46 | | |  | <.0001 | | |  | <.0001 | | |  | <.0001 | | |  | <.0001 | | | |  | | <.0001 |  |  |
| Somatic |  | .85 | | |  | <.0001 | | |  | <.0001 | | |  | <.0001 | | |  | <.0001 | | | |  | | <.0001 |  |  |
| Mental |  | .61 | | |  | <.0001 | | |  | <.0001 | | |  | <.0001 | | |  | <.0001 | | | |  | | .21 |  |  |
| Combined* |  | .50 | | |  | <.0001 | | |  | <.0001 | | |  | .0005 | | |  | <.0001 | | | |  | | .28 |  |  |
| Interaction |  | .78 | | |  | <.0001 | | |  | <.0001 | | |  | .18 | | |  | .0012 | | | |  | | .067 |  |  |
|  |  |  | | |  |  | | |  |  | | |  |  | | |  |  | | | |  | |  |  |  |

* Somatic and mental comorbidity
